# A truly human interface: interacting face-to-face with someone whose words are determined by a computer program

**DOI:** 10.3389/fpsyg.2015.00634

**Published:** 2015-05-18

**Authors:** Kevin Corti, Alex Gillespie

**Affiliations:** Department of Social Psychology, London School of Economics and Political Science, London, UK

**Keywords:** android science, cyranoid, dialog systems, embodiment, human–computer interaction, speech shadowing, Turing Test, uncanny valley

## Abstract

We use speech shadowing to create situations wherein people converse in person with a human whose words are determined by a conversational agent computer program. Speech shadowing involves a person (the shadower) repeating vocal stimuli originating from a separate communication source in real-time. Humans shadowing for conversational agent sources (e.g., chat bots) become hybrid agents (“echoborgs”) capable of face-to-face interlocution. We report three studies that investigated people’s experiences interacting with echoborgs and the extent to which echoborgs pass as autonomous humans. First, participants in a Turing Test spoke with a chat bot via either a text interface or an echoborg. Human shadowing did not improve the chat bot’s chance of passing but did increase interrogators’ ratings of how human-like the chat bot seemed. In our second study, participants had to decide whether their interlocutor produced words generated by a chat bot or simply pretended to be one. Compared to those who engaged a text interface, participants who engaged an echoborg were more likely to perceive their interlocutor as pretending to be a chat bot. In our third study, participants were naïve to the fact that their interlocutor produced words generated by a chat bot. Unlike those who engaged a text interface, the vast majority of participants who engaged an echoborg did not sense a robotic interaction. These findings have implications for android science, the Turing Test paradigm, and human–computer interaction. The human body, as the delivery mechanism of communication, fundamentally alters the social psychological dynamics of interactions with machine intelligence.

## Introduction

“Meaning is the face of the Other, and all recourse to words takes place already within the primordial face to face of language”

(Levinas, 1991, p. 206).

In comparison to other forms of interaction, face-to-face communication between humans is characterized by more social emotion, higher demands for comprehensibility, and increased social obligation; the face of the other commands an ethical relation that is absent in people’s interaction with “things” (Levinas, 1991). Face-to-face, close-proximity interaction between tangible bodies is the primordial human inter-*face* and is the format of exchange most conducive for shared understanding (Linell, 2009). Computer technologies specifically designed to simulate human social functioning (e.g., conversational agents) have to date communicated with people via technical interfaces such as screens, buttons, robotic devices, avatars, interactive voice response systems, and so on. This leaves a need to explore human perception of and interaction with these technologies under conditions that replicate the full complexity of face-to-face human–human communication. The present article introduces a means of doing so. We demonstrate a methodology that allows a person to interact “in the flesh” with a conversational agent whose interface is an actual human body.

## Contemporary Android Science

Android science aims to develop artificial systems identical to humans in both appearance and behavior (verbal and non-verbal) for the purposes of exploring human nature and investigating the ways in which these systems might integrate into human society (MacDorman and Ishiguro, 2006a; Ishiguro and Nishio, 2007). The field is as interested in better understanding people through their interacting with anthropomorphic technology as it is in further developing the technology itself. Considerable progress has been made in these endeavors, with perhaps the most notable work being that undertaken and inspired by Hiroshi Ishiguro of Osaka University’s Intelligent Robotics Laboratory, whose research and engineering teams have developed highly lifelike autonomous and semi-autonomous androids. MacDorman and Ishiguro (2006b) argue that in being controllable, programmable, and replicable, androids are in certain respects superior to human actors as social and cognitive experimental stimuli. They further contend that androids can evoke in humans expectations and emotions that attenuate the psychological barrier between people and machines.

The motor behaviors of autonomous androids are controlled by technologies that perceive and orient to the physical environment while their speech is controlled by a conversational agent. As autonomous technologies are still quite limited in terms of functionality, the social capacities of these types of androids are severely constrained. Tele-operated androids, meanwhile, overcome the limitations of fully autonomous models by-way-of a human operator controlling the android’s speech and movement (Nishio et al., 2007b). On account of their enhanced social capabilities, tele-operated androids have stimulated ample research in psychology and other domains of social and cognitive science. For instance, researchers have investigated the extent to which a person’s presence with remote others is amplified or weakened when tele-operating an android compared to when communicating in person or via more distal technological mediators such as video conferencing (Nishio et al., 2007a; Sakamoto et al., 2007). Researchers have also explored the extent to which tele-operators perceive their android to be extensions of themselves, sensing physical stimuli administered to the android as if the stimuli had been administered to their own body (Ogawa et al., 2012). Perhaps the most discussed phenomenon in the field of android science is the “uncanny valley,” posited by Mori (1970). This idea suggests that the affinity a person has for an artificial agent will increase as the appearance and motor behavior of the agent becomes more human-like; however, at a certain point along the human-likeness continuum (where the agent begins to look more or less human but for slight, yet telling, signs of artificiality) feelings of affinity will sharply decline, before rapidly rising again as the agent becomes indistinguishable from an actual human (MacDorman and Ishiguro, 2006b; Seyama and Nagayama, 2007).

We propose inverting the composition of tele-operated android systems in order to create hybrid entities consisting of a human whose words (and potentially motor actions) are entirely or partially determined by a computer program. We refer to such hybrids as “echoborgs,” which can be classified as a type of “cyranoid”— Milgram’s (2010) term for a hybrid composed of a person who speaks the words of a separate person in real-time. Echoborgs can be used to examine the role of the human body, as the delivery mechanism of communication, in mediating social emotions, attributions, and other interpersonal phenomena emergent in face-to-face interaction. Furthermore, echoborgs can be used to evaluate the performance and perception of artificial conversational agents under conditions wherein people assume they are interacting with an autonomously communicating human being. To ground these claims, however, we shall first discuss the tools and constraints of contemporary android science in order to identify where echoborg methodology can contribute.

### The Challenge of Creating Androids that Speak Autonomously

Examples of autonomous androids include Repliee Q1 and Repliee Q2, which were developed jointly by Osaka University and the Kokoro Corporation (see Ishiguro, 2005; Ranky and Ranky, 2005). Because androids of this nature attempt to replicate humans at both an outer/physical level as well as an inner/dispositional level, they can be evaluated against what Harnad (1991) defined as the *Total* Turing Test (also referred to as the Robotic Turing Test; Harnad, 2000), which establishes the entire repertoire of human linguistic and sensorimotor abilities as the appropriate criteria for judging machine imitations of human intelligence. The development of an autonomous android capable of passing such a test, however, remains a distant holy grail.

One source of current constraints concerns how artificial agents in general interpret and participate in dialog. Various terminologies describe technology that interacts with humans via natural language. “Dialog system,” “conversational agent,” and “conversational AI,” for instance, are terms used to denote the linguistic subsystems of artificial agents, though no clear consensus exists with regard to how non-overlapping these and other terms are. “Conversational agent,” the term we have employed thus far, is perhaps the most convenient term for conceptualizing the echoborg because it has been adopted by a parallel project—the development of embodied conversational agents (software that interfaces through onscreen anthropomorphic avatars). Much of the literature that distinguishes the functionality of various linguistic subsystems, however, couches these technologies as dialog systems. Types of dialog systems include high-level systems of integrated artificial intelligence that employ advanced learning and reasoning algorithms enabling a user and a machine to jointly accomplish specific tasks within a formal dialog structure (e.g., logistics and navigation planning agents), low-level systems that use basic algorithms to simply mimic, rather than understand, casual human conversation (e.g., web-based “chat bots”), and mid-level systems that strike a balance between high-level and low-level functionality (e.g., agents designed to field queries from and respond to pedestrians in transit centers; for a discussion of dialog system hierarchy, see Schumaker et al., 2007). Dialog systems can also be differentiated in terms of the level of initiative they take when interacting with users (Zue and Glass, 2000). System-initiative agents are those that control the parameters of dialog and elicit information from the user that must be compatible with certain response formats (e.g., interactive voice response telephone systems). User-initiative agents, on the other hand, are those in which the user presents queries to a passive agent (e.g., Apple’s Siri application). Mixed-initiative agents (by far the least developed variety; Mavridis, 2015) involve both the user and agent taking active roles in a joint task with the nature of dialog being qualitatively more conversational relative to other types of dialog systems.

If we treat, as Turing (1950) did, discourse capacity as a basic proxy for an interlocutor’s “mind,” then even today’s most advanced dialog system technologies render available to artificial agents such as androids minds that are at best starkly non-human (though potentially very powerful), and at worst extremely impoverished relative to that of humans. Though contemporary high-level and mid-level dialog systems are indeed impressive and their functionality continues to expand rapidly, they are not, in principle, attempts to mimic a human interlocutor capable of casual conversation. On the contrary, they are presently intended to interact with humans in specific domains and generally do not operate outside of these contexts (e.g., such a system cannot spontaneously switch from being a logistics planning agent to having a conversation about an ongoing basketball game). No human would be expected to communicate in a manner similar to these types of artificial intelligence, nor are humans necessarily constrained in terms of only being capable of communicating from within a fixed and narrow language-game. System-initiative and user-initiative agents also deviate from the norms of human–human interaction as they grant to one interlocutor total and unbreakable communicative control.

Though we can perhaps imagine high-level and mid-level dialog systems capable of engaging humans in casual conversation someday being ubiquitous throughout social robotics, at present only certain low-level and primarily text-based systems are engineered specifically for this purpose. An early but well known example of such a system is ELIZA, a chat bot with the persona of a Rogerian psychotherapist (Weizenbaum, 1966). Modern examples include A.L.I.C.E. (Artificial Linguistic Internet Chat Entity; Wallace, 2015), Cleverbot (Carpenter, 2015), Mitsuku (Worswick, 2015), and Rose (Wilcox, 2015). Many chat bots make use of the highly customizable AIML (Artificial Intelligence Markup Language) XML dialect developed by Wallace (2008) and operate by recognizing word patterns delivered by a user and matching them to response templates defined by the bot’s programmer. Increasingly sophisticated mechanisms for generating response corpora have been developed for chat bots in recent years. For instance, some developers have turned to real-time crowdsourcing of online communication repositories, such as Twitter and Facebook, as a means of producing responses appropriate for a given user input (see Mavridis et al., 2010; Bessho et al., 2012).

Chat bots are widely available on the internet and feature regularly in events such as the annual Loebner Prize competition (Loebner, 2008), a contest held to determine which chat bot performs most successfully on a Turing Test. This test involves a human interrogator simultaneously communicating via text with two hidden interlocutors while attempting to uncover which of the two is a bot and which is a real person. To date, no chat bot has reliably passed as a human being, and we are unlikely to see this feat accomplished in the near future (Dennett, 2004; French, 2012).

Generally, human interactions with chat bots fail to arrive at what conversation analysts refer to as “anchor points”: mutually attended to topics of shared focus that establish an implicit “center of gravity” during moments of conversation following routine canonical openings (Schegloff, 1986; Friesen, 2009). As chat bots tend to be user-initiative agents, they cannot engage in the type of fluid mixed-initiative conversation that is natural to mundane human–human interaction (Mavridis, 2015). Chat bots demonstrate a poor capacity to reason about conversation, cannot consistently identify and repair misunderstandings, and generally talk at an entirely superficial level (Perlis et al., 1998; Shahri and Perlis, 2008). According to Raine (2009), many chat bots work “based on an assumption that the basic components of a communication are on a phrase-by-phrase basis and that the most immediate input will be the most relevant stimulus for the upcoming output” (p. 399), an operative model that can lead conversation to irreparably fall apart when the perspectives of parties to a conversation diverge in terms of the meaning or intention each party assigns to an utterance. Human communication is fundamentally temporal and sequential, with many past and possible future utterances feeding into the meaning of a given utterance (Linell, 2009).

Developing acoustic technology that can accurately perceive spoken discourse remains a related challenge. The error rate of speech recognition technology is dramatically compounded by, among other things, variation in a speaker’s accent, the lengthiness and spontaneity of their speech, their use of contextually specific vocabulary, the presence of multiple and overlapping speakers, speech speed, and so on (Pieraccini, 2012). Thus, speech recognition systems within artificial agents perform best not when discerning casual conversational dialog, but when discerning brief and predictable utterances. Microphone array technologies and software capable of identifying and isolating multiple speakers continue to improve (e.g., the “HARK” robot audition system; Nakadai et al., 2010; Mizumoto et al., 2011), but demonstrations of these systems have essentially involved stationary apparatuses confined to laboratory environments.

### Tele-Operated Androids: Mechanical Bodies, Human Operators

Tele-operated androids were developed in part to overcome a social research bottleneck within android science born of the various limitations of conversational agents and perception technologies (Nishio et al., 2007b; Watanabe et al., 2014). They thus constitute a methodological trade-off: rather than being both physically artificial *and* having computer-controlled behavior (a combination that currently results in poor social functioning), the tele-operated paradigm cedes behavioral control to a human and in doing so augments the speech and motor capabilities of the android.

Perhaps the most well-known tele-operated android is Geminoid HI-1, a robot modeled in the likeness of its creator, Hiroshi Ishiguro. From a remote console, the tele-operator is able to transmit their voice through the geminoid (derived from the Latin word “geminus,” meaning “double”) while software analyzing video footage of the tele-operator’s body and lip movements replicate this motor behavior in the geminoid. The tele-operator can also manually control specified behaviors such as nodding and gaze-direction. Video monitors and microphones capture the audio-visual perspective of the geminoid and transmit to the tele-operation console, allowing the tele-operator to observe the geminoid’s social environment (Nishio et al., 2007b; Becker-Asano et al., 2010).

Relative to their fully-autonomous counterparts, the enhanced conversational capacities of tele-operated androids allow researchers to study communicatively rich human–android interactions as well as offer a means of operationally separating the behavioral control unit of an agent (the tele-operator) from the body, or interface, of the agent (the android). As Nishio et al. (2007b) contend:

“The strength of connection, or what kind of information is transmitted between the body and mind, can be easily reconfigured. This is especially important when taking a top-down approach that adds/deletes elements from a person to discover the “critical elements” that comprise human characteristics” (p. 347).

These methodological assets have inspired an abundance of exploratory laboratory and field work in recent years. Abildgaard and Scharfe (2012), for instance, used Geminoid-DK to conduct university lectures and reported on how perceptions of the android differed between male and female students. Research involving android-mediated conversations between parents and children has explored to what extent children sense the personal presence of a tele-operator (Nishio et al., 2008). Straub et al. (2010) studied how tele-operators and those they communicate with jointly construct the social identity of an android. Dougherty and Scharfe (2011), meanwhile, explored whether touch influences a person’s trust in a tele-operated android.

Despite the progress and promise of tele-operated androids, this line of research faces particular constraints. The non-verbal behaviors of autonomous and semi-autonomous androids are more mechanical and less fluid relative to humans. In their neuroimaging analysis of how people perceive geminoid movement, Saygin et al. (2012) show how incongruity between appearance (human-like) and motion (non-human-like) implicitly violates people’s expectations. Developing tools for matching an android’s bodily movements to those of its tele-operator is a major research priority (Nishio et al., 2007b), and improving techniques for achieving facial synchrony is particularly necessary given the intricate facial musculature of humans and the role of facial expression in conveying emotion and facilitating social interaction (Ekman, 1992; Bänziger et al., 2009; for a discussion of robot emotion conveyance, see Nitsch and Popp, 2014). Current anthropomorphic androids are relatively limited in terms of their capacity for human-like facial expressivity (Becker-Asano, 2011). For instance, Geminoid F’s face can successfully express the emotions *sad*, *happy*, and *neutral*, but the model struggles to convincingly convey *angry*, *surprised*, and *fearful* (Becker-Asano and Ishiguro, 2011). Also, the inexactness of an android’s lip movements in relation to the words spoken by its tele-operator has been discussed as possibly degrading the quality of social interactions (Abildgaard and Scharfe, 2012). Moreover, geminoids and other android models cannot walk on account of their having large air compressors facilitating numerous pneumatic actuators (Ishiguro and Nishio, 2007).

The imperfect appearance of tele-operated androids remains a barrier to replicating the social psychological conditions of face-to-face human–human interaction. Despite painstaking efforts to create realistic silicone android models (Ishiguro and Nishio, 2007), people are minutely attuned to subtle deviations from true humanness (e.g., eyes that lack glossy wetness). In a field study conducted to test whether people would notice an inactive or relatively passive geminoid in a social space, a majority of people reported having seen a robot in their surroundings (von der Pütten et al., 2011), a finding which suggests that most people are not easily fooled into believing an android is an actual person even in social situations where they do not engage the android directly. Moreover, though geminoids and other highly anthropomorphic androids are seen as the most human-like and least unfamiliar of robot types, people nonetheless perceive these androids as more threatening than less anthropomorphic models (Rosenthal-von der Pütten and Krämer, 2014).

There is also an important practical constraint characterizing the tele-operated and autonomous android paradigms. As Ziemke and Lindblom (2006) point out, it is quite time consuming and costly to produce android experimental apparatuses. This raises issues as to the scalability of the current android science research model and the extent to which experiments making use of a particular device in one laboratory can be replicated elsewhere.

## The Echoborg

An echoborg is composed of a human whose words (and potentially motor actions) are entirely or partially determined by a computer program. Echoborgs constitute a methodological trade-off inverse to that of the tele-operated paradigm discussed above, as they allow the possibility of studying social interactions with artificial agents that have truly human interfaces. The unique affordances of echoborgs can complement those of tele-operated and fully-autonomous androids and contribute to our understanding of the social psychological dynamics of human–agent interaction.

### Speech Shadowing and the Cyranoid Method

The echoborg concept stems from work conducted by Corti and Gillespie (2015), whose application of Milgram’s (2010) “cyranoid method” of social interaction demonstrates a means of creating hybrid human entities via an audio-vocal technique known as “speech shadowing.” Speech shadowing involves a person (the shadower) voicing the words of an external source simultaneously as those words are heard (Schwitzgebel and Taylor, 1980). This can be facilitated by-way-of an inner-ear monitor worn by the shadower that receives audio from the source. Research has shown that native-language shadowers can repeat the words of a source at latencies as low as a few hundred milliseconds (Marslen-Wilson, 1973, 1985; Bailly, 2003) and can perform the technique while simultaneously attending to other tasks (Spence and Read, 2003). Shadowers tend to reflexively imitate certain gestural elements of their source (e.g., stress, accent, and so on)—a phenomenon known as “phonetic convergence” (Goldinger, 1998; Shockley et al., 2004; Pardo et al., 2013).

**FIGURE 1:**
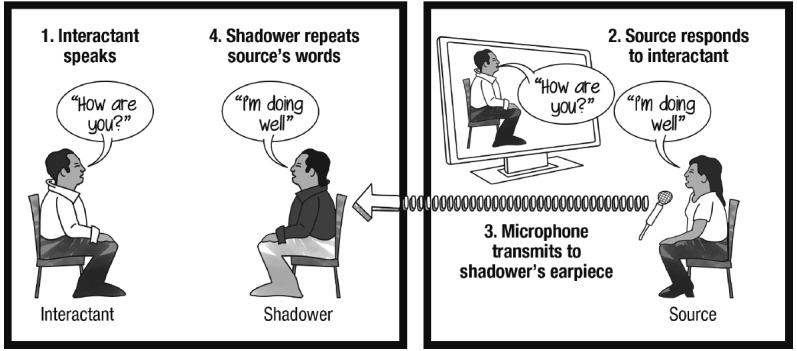
**Illustration of a basic cyranoid interaction.** The shadower voices words provided by the source while engaging with the interactant in person.

One finds the use of speech shadowing as a research tool primarily in psycholinguistics and the study of second-language acquisition. In the late 1970s, however, Milgram—famous for his controversial studies on obedience to authority (Milgram, 1974)—began using speech shadowing to investigate social scenarios involving people communicating through shadowers. He saw the technique as a means of pairing sources and shadowers whose identities differed in terms of race, age, gender, and so on, thus allowing sources to directly experience an interaction in which their outer appearance was markedly transformed (see Figure 1). From the point of view of the shadower, the method enabled exploration into the sensation of contributing to an unscripted conversation not one’s self-authored thoughts, but entirely those of a remote source. Inspired by the play *Cyrano de Bergerac*, the story of a poet (Cyrano) who assists a handsome but inarticulate nobleman (Christian) in wooing a woman by telling him what to say to her, Milgram referred to these source-shadower pairs as “cyranoids.”

As speech shadowing proved to be a relatively simple task that research participants were quick to grasp, Milgram quickly began exploring a variety of cyranic interactions. For instance, in several pilot studies he examined whether “interactants” (Milgram’s term for those who encountered a cyranoid) would notice if the source was changed mid-conversation (Milgram, 1977). Milgram (2010) also sourced for 11- and 12-year-old children during interviews with teachers naïve to the manipulation. Following these interactions, all of the teachers seemed to take the interviews at face value—they neither picked up on the true nature of the interactions nor sensed that the child they interviewed had behaved non-autonomously. The teachers had succumbed to the “cyranic illusion,” that is, the tendency to perceive interlocutors as autonomous communicators and thus fail to notice an interlocutor that is a cyranoid.

Corti and Gillespie (2015) argue that one of the cyranoid method’s primary strengths is that it allows the researcher to manipulate one component of the cyranoid, either the shadower or the source, while keeping the other component fixed. Thus, one can study how the same source is perceived when interacting through a variety of shadower-types. Conversely, a researcher can opt to keep the shadower constant and vary the identity of the source across experimental conditions. This capacity mirrors the functionality of tele-operated androids as well as similar methods for studying transformed social interactions (e.g., using 3D immersive virtual environment technology to alter people’s identities; see Blascovich et al., 2002; Bailenson et al., 2005; Yee and Bailenson, 2007). A unique benefit of the cyranoid method is that it allows for in person, face-to-face interactions between an interactant and a hybrid. When interacting with a cyranoid, one is not interacting with an onscreen person, or a human-like machine, or a virtual representation of a human, but with an actual human body.

While Corti and Gillespie’s (2015) recent work was conducted in the laboratory, it follows recent field explorations of cyranoids in experiential art installations (Mitchell, 2009) and as classroom learning tools (Raudaskoski and Mitchell, 2013). Taken together, these studies outline a number of basic protocols for constructing cyranic interactions and discuss the devices necessary for creating a basic cyranoid apparatus, which involves both a means of discreetly transmitting audio from the source to the shadower as well as a means for the source to hear (and, if possible, see) the interaction between the shadower and the interactant. The amalgam of devices one uses toward these requirements depends upon the type of interaction the researcher wishes to create. For instance, if a researcher wants to keep hidden from interactants the fact that a cyranoid is present in an interaction, then the cyranoid apparatus should be discreet and non-visible/audible to interactants. If the researcher wants the shadower to be mobile, then the devices that compose the cyranoid apparatus must transmit wirelessly. Minimizing the audio latency in the communication loop is crucial to any cyranoid apparatus; interactant→source and source→shadower audio transfer must be accomplished in a realistic amount of time.

A cyranic interaction involving a covert cyranoid is typically accomplished using an apparatus similar to the following. A wireless “bug” microphone placed near where the shadower and interactant engage each other transmits to a radio receiver listened to by the source in an adjacent soundproof room. The source speaks into a microphone connected to a short-range radio transmitter which relays to a receiver worn in the pocket of the shadower. Connected to the shadower’s receiver is a neck-loop induction coil worn underneath their clothing. The shadower wears a wireless, flesh-colored inner-ear monitor that sits in their ear canal and receives the signal emanating from the induction coil, allowing the shadower to hear and thus voice the source’s speech. This amalgam of devices is neither visible nor audible to interactants.

### Ceding Verbal Agency to a Machine

Echoborg methodology takes the original cyranoid model and replaces the human source with an artificial conversational agent. The words produced by the conversational agent are thus voiced and embodied by a human shadower. Echoborgs have at least four main research affordances:

#### Interchangeability of Shadowers and Conversational Agents

Both the shadower and the conversational agent that comprise an echoborg are easily customizable and interchangeable. The researcher need only train a confederate with the desired physical attributes to speech shadow sufficiently and then couple them with a conversational agent. This gives the researcher the freedom to construct many echoborgs, each differentiated from one another in terms their particular conversational agent, gender, age, and so on. Thus, one can observe how the same conversational agent is perceived depending on the identity of the shadower by holding the conversational agent constant across experimental conditions and varying the shadower (e.g., female shadower vs. male shadower). Alternatively, the researcher can hold the shadower constant and vary the conversational agent (e.g., ELIZA vs. A.L.I.C.E).

#### Visual Realism

Echoborgs offer a means of studying interactions under conditions where the interactant’s cognitive sense of the interaction is undistorted by any esthetic, acoustic, non-verbal, or motor non-humanness of the physical agent they encounter (e.g., lips that do not exactly align with the words they utter or eyes that do not perfectly make contact with the interactant’s). Speech shadowing is not a cognitively demanding task; it is rather simple for a well-rehearsed speech shadower to attend to other behaviors while replicating the speech of their source, including matching their body language to the words they find themselves repeating (e.g., shaking their head from side-to-side upon articulating the word “no”).

#### Mobility

Echoborgs can take advantage of the shadower’s physical mobility and need not be confined to stationary interactions—they can walk or otherwise move about while communicating with interactants. Human communication did not evolve for having conversations *per se*; it evolved for coordinating joint activity (Tomasello, 2008). Research on everyday language use shows that communication is a means of doing (Clark, 1996). Accordingly, mobile echoborgs open up the possibility of testing conversational agents in the context of performing a joint non-stationary activity.

#### Covert Capacity

Taking advantage of the cyranic illusion, echoborgs can interact with people covertly (i.e., under conditions wherein interactants assume they are encountering an autonomously communicating person). This affordance can be juxtaposed with the fact that at present, those who interact with tele-operated or autonomous androids are under no illusion that they are interacting with a fully-autonomous human being. The covert capacity of echoborgs thus presents a new means of researching interactions with conversational agents. It is one thing to evaluate interactions with conversational agents in contexts where people are cognitively aware, or at least primed to believe, that they are speaking to something artificial, but it is entirely different to study these systems under conditions where the interface one encounters (an actual human body) creates the visceral impression that one is dealing with an autonomous person.

## Overview of Studies

We conducted three experiments in which participants interacted with echoborgs. These studies explored the ways in which echoborgs, as human interfaces, mediate the experience of conversing with a chat bot in various contexts, as well as the extent to which echoborgs improve a chat bot’s ability to pass as human (i.e., be taken for a human rather than a robot). Each study was approved by an ethics review board at the London School of Economics and Political Science and conducted at the university’s Behavioral Research Laboratory. Adult participants were recruited online via the university’s research participant recruitment portal and included students from the university, university employees, and people unaffiliated with the university. Participants gave informed consent prior to participation and were debriefed extensively.

## Study 1: Turing Testing with Echoborgs

### Aims

In outlining the logic of his imitation game, Turing (1950) argued that “there was little point in trying to make a “thinking machine” more human by dressing it up in such artificial flesh” (p. 434) and made a clear distinction between what he thought of as the physical (likeness) and intellectual (functional) capacities of humans. However, this distinction has been criticized (Harnad, 2000); perceiving the salient bodily characteristics of other entities is fundamental to how humans infer the subjective states (or lack thereof) of said entities, be they real or unreal in reality (Graziano, 2013). To explore this tension, our first study investigated a Turing Test scenario wherein participants were asked to determine which of two shadowed interlocutors was truly human and which was a chat bot. Furthermore, we sought to determine whether a chat bot voiced by a human shadower would be perceived as more human-like than the same bot communicating via text.

### Shadowers and Subjects

Two female graduate students (both aged 23) were trained as speech shadowers. Eighty-two participants (42 female, mean age = 28.93, SD = 12.05) were randomly assigned into pairs within one of two experimental conditions: Text Interface (*n* = 21) and Echoborg (*n* = 20). One participant within each pair was randomly selected to function as the Turing Test interrogator while the second participant was designated as the human interlocutor. In all pairs, participants were both unfamiliar with one another and unaware of the other’s role in the study.

### Procedure

From the interaction room, the researcher instructed the interrogator that the study involved using a text-based instant messaging client (Pidgin) to simultaneously communicate with two anonymous interlocutors, one of whom was a chat bot (Cleverbot). The interrogator’s computer showed two separate text-input windows, one that delivered to “Interlocutor A,” and another that delivered to “Interlocutor B.” The interrogator was told that following 10-min of conversation they would be asked which of these two interlocutors they believed was the real human. Meanwhile, in a separate room, a research assistant instructed the human interlocutor that the study involved holding a 10-min conversation with a stranger and that their task was to simply respond to messages that appeared on a computer screen. The human interlocutor was thus blind to the fact that they were engaged in a Turing Test. Both the interrogator and the human interlocutor were informed that they were free to discuss any topic during the interaction so long as nothing was vulgar.

#### Text Interface Condition

Once instruction was complete, the researcher relocated to a third room (the source room) where they monitored the interaction using a computer. Messages that the interrogator typed to Interlocutor A were routed to the researcher, who input the received text into Cleverbot and routed Cleverbot’s response back through the instant messaging client to the interrogator. Messages the interrogator sent to Interlocutor B, meanwhile, were routed to the human interlocutor’s computer, and the human interlocutor directly responded in text via the instant messaging client.

#### Echoborg Condition

The interrogator was further instructed that though they would type messages to Interlocutor A and Interlocutor B via the instant messaging client, the responses of these two interlocutors would be spoken aloud by two speech shadowers. The two speech shadowers, with shadowing equipment, entered the room, sat side-by-side facing the interrogator at a distance of roughly six feet, and it was made known to the interrogator which shadower would reproduce the words of Interlocutor A and which would reproduce the words of Interlocutor B (shadowers alternated between trials in terms of the interlocutor they were paired to). The interrogator was informed that the shadowers would speak solely words they received from their respective sources and that at no point during the interaction would the shadowers speak self-authored thoughts. Furthermore, the interrogator was informed that both interlocutors would only respond to typed messages and that nothing the interrogator spoke aloud would be responded to.

**FIGURE 2:**
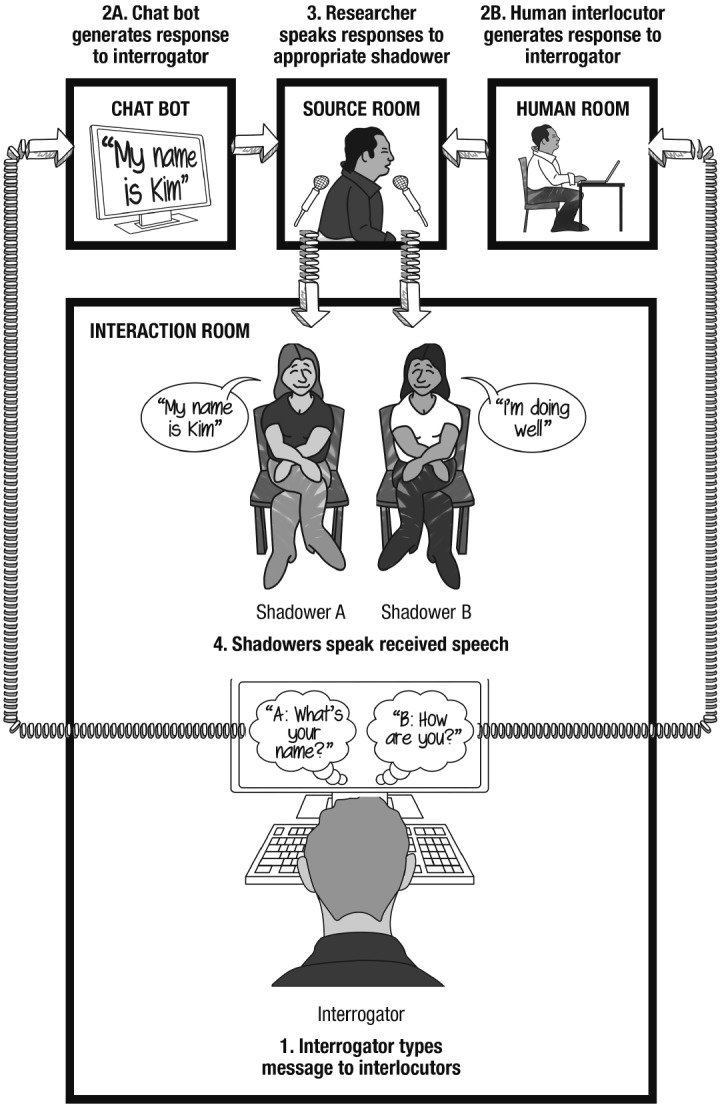
**Illustration of a Turing Test scenario involving speech shadowing.** This figure visually depicts the Echoborg condition in Study 1.

Following these instructions, the researcher relocated to the source room. As in the Text Interface condition, messages that the interrogator sent to Interlocutor A were routed to the researcher’s computer where they were input by the researcher into Cleverbot. Instead of routing Cleverbot’s responses back to the interrogator through the instant messaging client, however, the researcher spoke Cleverbot’s responses into a microphone which relayed to the speech shadower paired to Interlocutor A, thus allowing them to hear and repeat Cleverbot’s words to the interrogator. Similarly, the human interlocutor’s typed responses were routed to the researcher’s computer (rather than directly to the interrogator), allowing the researcher to speak these messages into a separate microphone which relayed to the shadower paired to Interlocutor B (see Figure 2).

#### Stock Responses

Cleverbot’s response formats are not programmed; Cleverbot references past conversations it has held with people over the internet when generating a reply to a given user input (Carpenter, 2015). Unlike other bots, therefore, Cleverbot has no consistent identity. Its strength lies in its ability to learn unique ways of responding. We decided, however, that in order to establish consistency between experimental trials, three stock responses would be supplied in both conditions to the interrogator in lieu of a response generated by Cleverbot. Each time the interrogator inquired as to the name of Interlocutor A, the standard response “My name is Kim” was supplied to the interrogator. In response to questions as to what Interlocutor A’s occupation was, the response “I’m a psychology student here” was supplied. Finally, in response to questions concerning where Interlocutor A was from, the response “I’m from London” was given.

### Measures

Following the interaction, the interrogator indicated on a questionnaire which of the two interlocutors (A or B) they believed was the real human and indicated along a 10-point scale how confident they were that they had made the correct identification (1: not at all confident; 10: highly confident). Interrogators also rated each interlocutor along a 10-point scale in terms of how human-like they seemed (1: seemed very mechanical and computer-like; 10: seemed very human-like).

### Results

In the Text Interface condition, 21 out of 21 interrogators correctly identified Interlocutor B as being the real human, compared to 18 out of 20 interrogators in the Echoborg condition, a non-significant difference, *z* = 1.49, *p* = 0.14 (two-tailed). There was no significant difference between conditions in terms of how confident interrogators were with regard to their answers, with interrogators in the Text Interface condition reporting an average confidence of 7.67 (SD = 2.61) and interrogators in the Echoborg condition reporting an average confidence of 7.55 (SD = 1.70), *t*(39) = 1.68, SE = 0.69, *p* = 0.87.

Human-likeness ratings were compared using a repeated measures analysis of variance, with *Condition* (Text Interface vs. Echoborg) treated as a between-subjects factor and *Interlocutor* (Interlocutor A vs. Interlocutor B) treated as a within-subjects factor. There was a significant main effect of *Interlocutor* showing that Interlocutor B was perceived as significantly more human-like than Interlocutor A in both conditions, *F*(1,39) = 130.87, *r* = 0.88, *p* < 0.001. There was also a significant interaction between *Condition* and *Interlocutor*, *F*(1,39) = 7.23, *r* = 0.40, *p* < 0.05. Independent samples means tests showed that the average human-likeness rating of Interlocutor A in the Text Interface condition (*M* = 2.14, SD = 1.15) was significantly less than the average rating in the Echoborg condition (*M* = 4.05, SD = 2.42), *t*(39) = –3.25, SE = 0.59, *p* < 0.01. Meanwhile, the average human-likeness rating of Interlocutor B in the Text Interface condition (*M* = 8.76, SD = 1.51) was not significantly different from the average rating in the Echoborg condition (*M* = 8.15, SD = 1.46), *t*(39) = 1.32, SE = 0.46, *p* = 0.20.

### Discussion

The interface (human body vs. text) engaged by the interrogator made no statistically significant difference in terms of their ability to discern which interlocutor was the real human. The chat bot, however, was perceived by interrogators as significantly more human-like when being shadowed by a person compared to when simply communicating via text. This contrasted with the fact that how human-like human interlocutors seemed to participants did not depend on whether their words were voiced by a speech shadower. This suggests that as the quality of an interlocutor’s discourse capacity improves (i.e., becomes more human) in Turing Test scenarios, the role the interface plays in eliciting judgments about human-likeness declines.

## Study 2: A Human Imitating a Chat Bot?

### Aims

Study 2 investigated whether attributing human agency to an interlocutor is increasingly determined by the nature of the interface as the words spoken by the interlocutor provide less definitive evidence. We designed a scenario wherein participants encountered an interlocutor and had to determine whether the interlocutor was (a) a person communicating words that had been generated by a chat bot, or (b) a person merely imitating a chat bot, but nonetheless speaking self-authored words (the former option always being true). The point here was to see whether or not the interface participants encountered (human body vs. text) influenced whether they thought their interlocutor was producing self-authored words or, alternatively, those of a machine. The framing of the scenario leads participants to expect that the communication offered by their interlocutor will be abnormal, thus the conversational limitations of chat bots are not a liability as they are in standard Turing Test scenarios. By design, participants must form an attribution regarding the communicative agency of their interlocutor under conditions of ambiguity.

Research on perceptual salience suggests that people will deem causal what is salient to them in the absence of equally salient alternative explanations (Jones and Nisbett, 1972; Taylor and Fiske, 1975). Dual process information evaluation theories propose that when a person evaluates the communication and behavior of others, stimulus ambiguity increases reliance on heuristic cues (e.g., appearance) at the expense of more thoughtful situational evaluation (Sager and Schofield, 1980; Devine, 1989; Chen and Chaiken, 1999). We extrapolated from this research that when faced with an ambiguous situation in which one’s interlocutor was either truly speaking words generated by a chat bot or merely pretending to be one, the interface (and thereby the heuristic cues) salient to the participant would determine how they attributed authorship to the words they encountered. We therefore hypothesized that those who encountered an echoborg would be more likely to see their interlocutor as producing self-authored words (imitating a chat bot) compared to those who encountered an interlocutor through a text interface.

### Shadowers and Subjects

A female graduate student (aged 30) was trained to perform as a speech shadower. Fifty-eight adult participants (35 female; mean age = 25.19, SD = 9.08) were randomly assigned to one of two conditions: Echoborg (*n* = 28) and Text Interface (*n* = 30).

### Procedure

As with Study 1, Cleverbot, as well as the three stock responses described above, were used in all trials.

The participant was led to an interaction room and instructed by the researcher that the study involved holding a 10-min conversation with an interlocutor who was either (a) communicating solely words that had been generated by a chat bot program (at no point speaking anything self-authored), or (b) simply imitating a chat bot program, but producing self-authored words nonetheless. The researcher ensured that the distinction between these scenarios was clear to the participant and gave the further instruction that the participant would be asked following the interaction which of the two scenarios they believed to have been the case. The participant was informed that they were free to discuss anything they liked with their interlocutor so long they refrained from vulgarity.

Unlike Study 1, which had participants send messages to their interlocutors via an instant messaging client, Study 2 featured participants speaking aloud to their interlocutor as they would during any other face-to-face encounter, thereby increasing the mundane realism of the scenario. The apparatus for this type of interaction, however, required a means of inputting the participant’s spoken words into the chat bot in the form of text. As we deemed speech-to-text software to be insufficient for our purposes (being too slow and inaccurate), we settled on a procedure wherein the researcher (from an adjacent room) acted as the chat bot’s ears and speed typed the participant’s words into the chat bot as they were being spoken, paraphrasing when necessary for particularly verbose turns. This can be conceptualized as a minimal technological dependency format of the echoborg method (as opposed to a full technological dependency format which would place acoustic perception solely on technology). Although a minimal technological dependency format adds an additional human element to the communication loop, it ensures that accurate representations of interactants’ words are processed by the conversational agent.

#### Text Interface Condition

**FIGURE 3:**
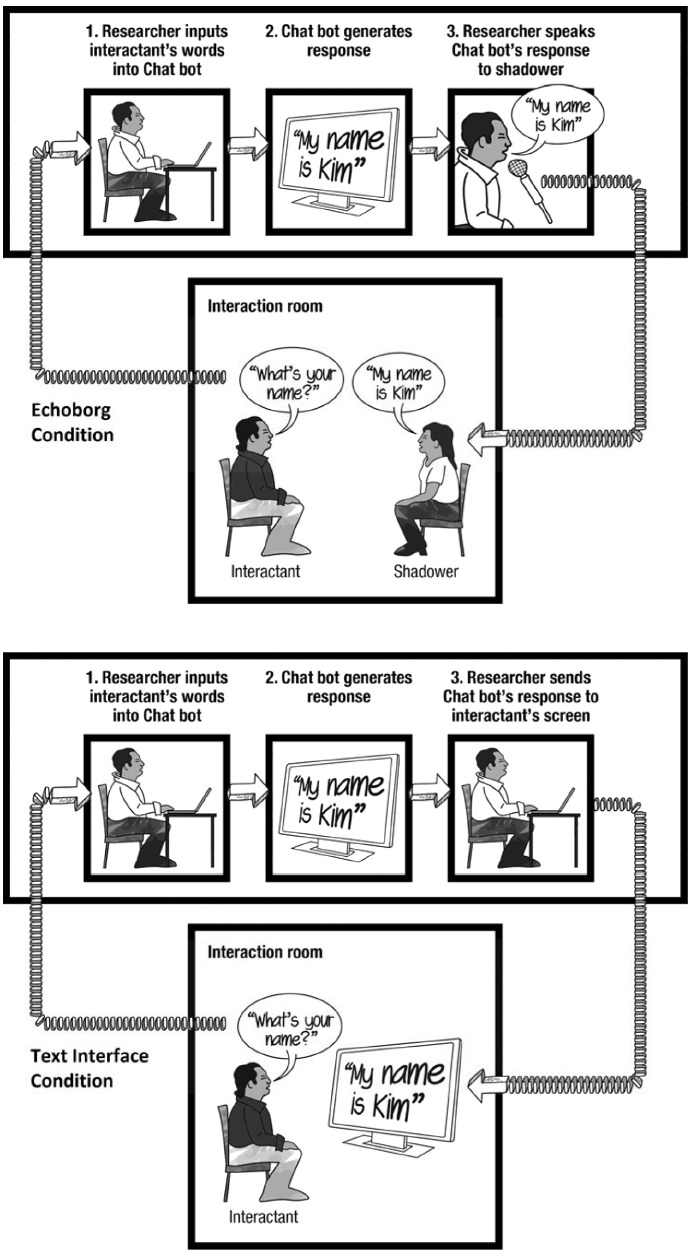
**Illustration of interaction scenarios in Study 2 and Study 3**.

The participant was seated in front of a computer screen which displayed a blank instant messaging client chat window. The participant was instructed that they were to address their interlocutor by speaking aloud and that their interlocutor would respond via text readable in the chat window. Once instruction was complete, the researcher left the interaction room and returned to the adjacent source room. From the source room, the researcher overheard words spoken by the participant via a covert wireless microphone and speed typed them into Cleverbot’s text-input window. Cleverbot’s responses were then sent through the instant messaging client to the participant’s screen in the interaction room (see Figure 3).

#### Echoborg Condition

The participant was instructed that as soon as the researcher left the interaction room their interlocutor would enter and sit facing the participant (at a distance of roughly six feet). The participant was not made aware of the fact that their interlocutor would be wearing an earpiece and receiving messages via radio, and the cyranoid apparatus was not visible to the participant. The researcher then left the interaction room and returned to the adjacent source room while the shadower entered the interaction room and sat across from the participant. The researcher listened to the words of the participant via a covert wireless microphone, speed typed them into Cleverbot’s text-input window, and subsequently spoke Cleverbot’s responses into a microphone which relayed to the shadower’s inner-ear monitor.

### Measures

Following the interaction, the participant indicated on a questionnaire whether they thought their interlocutor had truly been producing words generated by a chat bot program or whether their interlocutor was simply imitating a chat bot.

### Results

Of the 30 participants in the Text Interface condition, 11 stated following the interaction that they believed their interlocutor was simply imitating a chat bot compared to 22 of 28 participants in the Echoborg condition. A binary logistic regression model showed these proportions to be significantly different from one another, OR = 6.33, *b* = 1.85, SE = 0.60, *p* < 0.01 (indicating that the odds of a participant in the Echoborg condition deciding their interlocutor was imitating a chat bot were 6.33 times greater than the odds of a participant in the Text Interface condition coming to the same conclusion).

To gain a sense of the audio latency dynamics of echoborg interactions involving minimal technological dependency, we randomly selected four trials from the Echoborg condition and measured the time between the conclusion of each interactant-utterance and the commencement of the echoborg’s subsequent response. The average latency was 5.15 s (SD = 3.04 s).

### Discussion

Our results indicate that under conditions of ambiguity wherein the source of an interlocutor’s verbal agency is unclear, the interface substantially affects whether one attributes human agency to the words one’s interlocutor produces. Participants who communicated with a chat bot via a text interface were significantly more likely to see their interlocutor as actually producing words generated by a chat bot compared to those who encountered the same chat bot but through a human shadower. The results from this study corroborate the notion that the cyranic illusion is robust in circumstances involving extreme source-shadower incongruity: people are biased toward perceiving an echoborg as an autonomous person.

Our findings suggest that it is relatively easy to get a chat bot to be perceived as an autonomous human if one is free to manipulate the contextual frame (i.e., the social psychological context of the interaction). An ostensibly simple suggestion from the experimenter (i.e., that an interlocutor might be a human imitating a chat bot) can shift the entire contextual frame, fundamentally altering attributions of agency. Indeed, whenever it is claimed a certain bot has “passed the Turing Test” or some variant of Turing’s game, it usually has less to do with advances in conversational agent technology and more to do with shifting the contextual frame (e.g., when the chat bot Eugene Goostman—a bot that poses as a 13-year-old Ukrainian boy with limited English skills and general knowledge—was declared as having successfully fooled 33% of interrogators in a Turing Test in 2014; You, 2015). This, however, raises a fundamental question: within what contextual frame *should* participants encounter chat bots when we evaluate them? Arguably, the most important frame is the most common, namely, the everyday assumption that our interlocutors are human, just like us.

## Study 3: Can Covert Echoborgs Pass as Human in the Everyday Contextual Frame?

### Aims

Study 3 examined people’s impressions following their conversing with an agent who, unbeknownst to them, produced solely the words of a chat bot. We aimed to gauge whether or not being shadowed by a human improved a chat bot’s ability to pass as an actual person within the everyday contextual frame (i.e., under the conditions of a generic social encounter wherein it is assumed an interlocutor is an ordinary human). The concept of “passing” within such a frame comes from the sociological and social psychological traditions that explore the mechanisms through which people manage identities in order to be accepted as a member of a particular group (Goffman, 1963; Renfrow, 2004; Khanna and Johnson, 2010). For example, the anthropomorphic androids in Dick’s (1968) novel *Do Androids Dream of Electric Sheep?* were able to pass as human so long as they concealed their true nature, took part in mundane human activities, and avoided the scrutiny of bounty hunters. The speech shadower in an echoborg is essentially a human mask placed over the peripherals one normally associates with computer systems. From a static third-person point of view, therefore, echoborgs appear to be autonomous human beings and nothing more, raising the question as to whether or not despite their communicative deficiencies people still sense that echoborgs are ordinary people. We predicted that research participants would not leave an interaction with a covert echoborg with the impression of having communicated with something non-human, whereas interacting with a covert chat bot through a text interface would leave participants with a strong impression of having encountered machine intelligence of some sort.

This study also investigated perceptual phenomena associated with the uncanny valley, namely how human-like, eerie, and familiar a covert echoborg interlocutor would seem to those with whom they communicated, and whether or not people would be comfortable in the presence of a covert echoborg. Mori’s (1970) original hypothesis suggested that “subtle deviations from human appearance and behavior create an unnerving effect” (MacDorman and Ishiguro, 2006b, p. 299), and our goal was to gauge people’s reaction to an interlocutor that was human in all respects but for the fact that a conversational agent determined the words they spoke.

### Shadowers and Subjects

A female graduate student (aged 23) was trained to perform as a speech shadower. Forty-one adult participants (26 female; mean age = 24.12, SD = 7.59) were randomly assigned to one of two conditions: Echoborg (*n* = 20) and Text Interface (*n* = 21).

### Procedure

In addition to Cleverbot, two other chat bots were used in this study: Mitsuku (winner or the 2013 Loebner Prize) and Rose (winner of the 2014 Loebner Prize). In the Echoborg condition, Cleverbot and Rose were each assigned to speak with seven participants while Mitsuku spoke with six participants. In the Text Interface condition, Cleverbot, Rose, and Mitsuku each spoke with seven participants. During Cleverbot trials, the stock responses used in the prior two studies were employed.

The participant was instructed that the study concerned how strangers conversed when speaking for the first time, that it involved simply holding a 10-min conversation with another research participant, and that they were free to decide on topics for discussion so long as vulgarity was avoided. The researcher made no mention of chat bots or of anything related to artificial intelligence. Furthermore, the participant was given no indication that their interlocutor would behave non-autonomously or abnormally. The aim was to invoke the everyday contextual frame, in so far as that can be done within an experimental setting.

This study used the same minimal technological dependency apparatus and procedure as in Study 2. In the Text Interface condition the participant spoke aloud to their interlocutor while their interlocutor’s responses were shown in text on a computer screen. In the Echoborg condition the participant encountered a human shadower face-to-face.

### Measures and Post-Interaction Interview

Following the interaction the participant completed a brief questionnaire containing items asking them to indicate on a 10-point scale how human-like (1: very mechanical and computer-like; 10: very human-like), eerie (1: not at all eerie; 10: very eerie), and familiar (1: not at all familiar; 10: very familiar) their interlocutor seemed, as well as how comfortable they felt during the interaction (1: not at all comfortable; 10: very comfortable). Participants were also asked to briefly describe in writing the person they spoke with and what they thought they study was about.

When the questionnaire was completed, the researcher interviewed the participant to gain a sense of their impressions of the interaction and their interlocutor. The participant was asked to describe salient aspects of their interlocutor’s personality. In order to ascertain whether the participant had picked up on the fact that they had communicated with a computer program, the researcher asked the participant whether they had suspicions regarding the nature of their interlocutor or about the study generally. Finally, the researcher revealed to the participant the full nature of the interaction and disclosed the purpose of the study.

### Results

In the Text Interface condition, 14 of 21 participants (67%) mentioned during their post-interaction interview (prior to the researcher making any allusion to chat bots or anything computer-related) that they felt they had spoken to a computer program or robot. Two participants stated during debriefing that they suspected their interlocutor was a real person acting or using a script. Furthermore, seven participants (33%) explicitly stated in writing on their questionnaires that they believed the purpose of the study was to assess human–computer/human–robot interaction. Of the 14 participants who did not indicate that they thought the purpose of the study involved human–computer interaction, six said that they thought the study concerned how strangers communicated with one another (the stated purpose of the study supplied by the researcher prior to the interaction). Two participants believed the study concerned how people handle abnormal/unexpected situations. Six participants provided unique responses that did not fit into these categories.

Only 3 of 20 participants (15%) in the Echoborg condition stated during their post-interaction interview that they felt as though they had spoken to a computer or robot. Fifteen participants made it clear to the researcher during their interview that they suspected their interlocutor had been acting or giving scripted responses that did not align with their actual persona. Only two participants (10%) indicated in writing on their questionnaires that they believed the purpose of the study was to assess human–computer/human–robot interaction. Of the 18 participants who did not indicate that they thought the study’s purpose was to investigate human–computer interaction, only one stated that they thought the purpose of the study was to investigate communication between strangers. Seven participants believed the purpose of the study related to how people deal with abnormal/unexpected situations (e.g., “how people react when thrown out of their comfort zone” and “how people react to people who do not comply with social norms”). Four participants believed the study’s purpose was to see how people communicated those who were shy/introverted. Three participants stated that they thought the study’s purpose involved how people communicate with those who have a disability such as autism or speech impairment. Four participants provided other unique responses.

We performed a multivariate analysis of variance to see whether *Interface* (Echoborg vs. Text Interface) and *Chat Bot* (Cleverbot vs. Mitsuku vs. Rose) produced effects on participants’ judgments concerning the four questionnaire items that pertained to how familiar, eerie, and human-like their interlocutor seemed as well as how comfortable they felt during the interaction. An initial omnibus test showed a significant effect of *Interface*, Λ = 0.73, *F*(4,34) = 3.18, *p* < 0.05, η^2^ = 0.27, and a non-significant effect of *Chat Bot*, Λ = 0.74, *F*(8,68) = 1.41, *p* = 0.21, η^2^ = 0.14. Univariate tests showed a significant effect of *Interface* on how comfortable participants felt during the interaction, *F*(1,37) = 10.64, *p* < 0.01, η^2^ = 0.22, with participants in the Text Interface condition reporting higher levels of comfort (*M* = 5.52, SD = 2.42) compared to those in the Echoborg condition (*M* = 3.44, SD = 2.04). However, these univariate tests showed non-significant effects of *Interface* with respect to how familiar, *F*(1,37) = 1.52, *p* = 0.23, η^2^ = 0.04, eerie, *F*(1,37) = 0.08, *p* = 0.77, η^2^ < 0.01, and human-like, *F*(1,37) = 0.24, *p* = 0.63, η^2^ = 0.01, interlocutors seemed. In the Text Interface condition, mean scores for familiarity, eeriness, and human-likeness were 3.81 (SD = 1.89), 6.19 (SD = 2.14), and 2.95 (SD = 1.63), respectively, compared to scores of 3.00 (SD = 2.22), 6.00 (SD = 2.00), and 2.70 (SD = 1.78), respectively, within the Echoborg condition.

Two Echoborg condition trials for each chat bot were selected at random and the audio latency was assessed. The average latencies for Cleverbot, Mitsuku, and Rose were 4.43 s (SD = 2.92 s), 5.95 s (SD = 3.98 s), and 3.96 s (SD = 3.94 s), respectively. As each trial made use of the same minimal technological dependency format of interaction, the differences between these latencies can be accounted for by the fact that the chat bots we used differ in terms of the speed at which they generate and return responses.

### Discussion

In line with our hypothesis, a majority of participants in the Text Interface condition sensed they were communicating with a chat bot despite being led to believe they would be talking to another research participant while only a small minority of participants in the Echoborg condition came to the same conclusion. These results suggest that a chat bot stands a far greater chance of passing as a human in an everyday contextual frame when being shadowed by a human than when communicating via a text interface. The caveat to these findings, however, is that interactants do not tend to see a person shadowing for a chat bot as genuine. Rather, interactants see such people as deliberately behaving outside of their normal persona. This finding corroborates the general phenomenon observed in Study 2, that people are inclined to perceive an echoborg as somebody acting but nonetheless speaking self-authored words. We should note, however, that participants’ awareness of being in a laboratory study may have contributed to their suspecting that the persona they encountered was not genuine. Future research may include observational field studies wherein interactants encounter a covert echoborg in real-world social contexts (e.g., a generic social gathering). It is plausible that in such scenarios interactants would be less inclined to form the belief that an echoborg was someone deliberately acting outside of their normal persona.

Although our experiment only considered two types of interfaces as opposed to a continuum of interfaces ranging from the very-human to the very-mechanical, our results contribute a novel finding to the discussion surrounding uncanny valley phenomena. We found evidence that people feel significantly less comfortable speaking to a chat bot through a human speech shadower than they do speaking to the same chat bot through a text interface. General discomfort seemed to derive from the social awkwardness that arose due to the chat bot’s violations of conversational norms. The effect of these violations appears to have been magnified in the Echoborg condition. It is likely that participants in the Echoborg condition held higher expectations about the level of understanding and rapport that would be reached and sustained during the interactions on account of their speaking face-to-face with another human being, for the physical body of the other is laden with social cues that evoke such expectations (Kiesler, 2005). Komatsu and Yamada’s (2011) “adaptation gap” hypothesis suggests that when expectations are not met during interactions with agents (e.g., when the implied social capacity of an agent exceeds that actually experienced by a user), people’s subjective impressions are affected. Accordingly, participants in the Echoborg condition may have felt more uncomfortable compared to their counterparts in the Text Interface condition partly due to their having higher pre-interaction expectations about the quality of interlocution they would experience. What requires further study is the investigation of conditions within which participants are told prior to interacting with either an echoborg or a text interface that their interlocutor will be producing the words of a chat bot. Adding two such conditions to Study 3′s design would allow one to observe whether the body of the other produces effect on feelings of comfort independent of pre-interaction expectations.

## General Discussion

We have introduced and demonstrated a new research method, a special type of cyranoid we call an echoborg. Echoborgs make possible interactions with artificial conversational agents that have truly human interfaces. Though an abundance of research has demonstrated various means of embodying machine intelligence in human form, from onscreen embodied conversational agents (e.g., Cassell et al., 2000; Krämer et al., 2009) to 3D agents in immersive virtual environments (e.g., Selvarajah and Richards, 2005; Bailenson et al., 2008) to tangible machine-bodied androids (e.g., Ishiguro and Nishio, 2007; Spexard et al., 2007), the echoborg stands apart from these other methods in that it involves a real, tangible human as the interface.

Study 1 compared a standard text-based version of the Turing Test to an echoborg version and found that although a chat bot’s ability to pass a Turing Test was not improved when being shadowed by a human, being shadowed did increase ratings of how human-like the chat bot seemed. This effect of embodiment on human-likeness was unique to chat bot interlocutors, as human interlocutors in these tests were not seen as more human-like when their words were spoken by a human shadower, suggesting that a demonstrated capacity for human-level dialog may override the effect of human embodiment on perceptions of human-likeness in Turing Test contexts. Study 2 showed that in an ambiguous situation wherein participants were told that an interlocutor was either articulating words generated by a chat bot or merely imitating one, participants in a text interface condition were more likely to conclude that they had encountered the words of an actual chat bot than those who encountered an echoborg. The contrast between these two conditions provides evidence for (a) the robustness of the cyranic illusion, and (b) the notion that people’s causal attributions align with what is most salient and least ambiguous to them. Study 3 explored the notion of passing and the uncanny valley in an ordinary, everyday contextual frame (i.e., the experimental context attempted to simulate a generic, unscripted, first-time encounter between strangers). Participants engaged with a covert chat bot via either a text interface or an echoborg. When interviewed following these interactions, most of the participants who engaged a text interface suspected they had encountered a chat bot, whereas only a few of the participants who engaged an echoborg held the same suspicion. This suggests that it is possible for a chat bot to pass as fully human given the requisite interface, namely an actual human body, and a suitable contextual frame. This study also found that people were less comfortable speaking to an echoborg than to a text interface.

### Implications

#### Android Science

**FIGURE 4:**
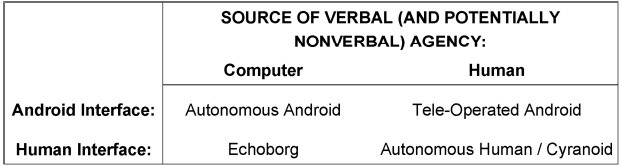
**Basic tools of android science**.

Drawing from Nunamaker et al.’s (2011) distinction between virtual avatars and embodied conversational agents, in Figure 4 we visualize a simple two-dimensional matrix differentiating the basic tools available to android science, with one dimension indicating the source of verbal (and potentially non-verbal) agency and the other indicating interface-type. This matrix places the echoborg in relation to current mechanical devices utilized by android researchers (autonomous and tele-operated androids) as well as human beings as experimental subjects. By juxtaposing the field’s tools in this manner, we can begin formally distinguishing the unique research questions that lend themselves to each. The fundamental question that each of these tools can be applied to concerns what happens when the human elements of an interlocutor are removed and replaced by artificial imitations. The unique questions that can be approached via the usage of echoborgs concern how real human bodies (not mere mechanical imitations) fundamentally alter people’s perceptions of and interactions with machine intelligence.

In the echoborg paradigm, the communicative limitations of chat bots and other types of conversational agents are not treated as problematic barriers to fluid conversation. Rather, these limitations are directly operationalized; how the human body as an interface mediates the perception of these communicative limitations is what is of interest. We can thus differentiate the echoborg paradigm from the tele-operated android paradigm in the following manner. Tele-operated android research targets the social dynamics between humans and human-like machine interfaces. Given that conversational agents are relatively poor communicators, the tele-operated paradigm cedes speech-interpretation/generation responsibility to a human operator, whose experiences operating an android can also be the subject of inquiry. By contrast, the echoborg paradigm is interested in the social dynamics that emerge when the words artificial systems produce are refracted through actual human bodies during face-to-face interaction.

The affordance which grants the echoborg particular promise as a methodology is that it allows researchers the opportunity to study interactions under conditions wherein people believe they are speaking to an autonomously communicating person. The echoborg can interact covertly (i.e., without interactants expecting that they are communicating with a bot). Of course, chat bots and other conversational agents can be deployed covertly via traditional text interfaces—and many are (e.g., posing as real people in chat rooms, web forums, and social media websites in order to distribute marketing messages and collect user-data; Gianvecchio et al., 2011; Nowak, 2012). But as Study 3 shows, focused interaction with a covert chat bot via a text interface for a sustained period of time is very likely to result in the interactant sensing that that they are not speaking to an actual person. Today’s chat bots simply fail to sustain meaningful mixed-initiative dialog, and unless their words are vocalized by a tangible human body, their true nature is quickly exposed.

#### The Turing Test Paradigm (and Passing)

Over half a century since its conception, the Turing Test paradigm remains a substantial area of interest in artificial intelligence and philosophy of mind. The usefulness of the Turing Test as a technological benchmark, its rules, and what it would mean for a machine to pass such a test (i.e., what, exactly, passing would be evidence of) are issues that have been hotly debated (e.g., Searle, 1980; Copeland, 2000; French, 2000; Harnad, 2000; Chomsky, 2008; Watt, 2008; Proudfoot, 2011). The non-philosophical literature on the Turing Test focuses largely on the technological aspects of candidate conversational agents (e.g., whether they occasionally make spelling mistakes) and the conditions that give rise to increased fooling (e.g., knowing vs. not knowing of the possible presence of a machine intelligence; Saygin and Cicekli, 2002; Gilbert and Forney, 2015). What remains to be explored in sufficient depth are the social psychological dynamics within standard and modified Turing Test scenarios: causal attributions, identity and power relationships, questions asked and avoided, misunderstandings recognized and repaired, intersubjective achievement, and so on (e.g., Warwick and Shah, 2015). Our position is that the Turing Test is most useful when its orthodox interpretation is relaxed and it is applied not toward assessing the capacities of chat bots *per se*, but toward investigating aspects of human social nature. Indeed, the chat bot itself may be the *least* interesting element within a Turing Test scenario. A chat bot can be made to fool a human interrogator if the expectations of the interrogator are manipulated (e.g., through ambiguous framing). What is interesting is exploring the ways in which the chat bot’s utterances interact with the interrogator’s expectations, all within a particular contextual frame, so as to produce a social interaction that feels more or less comfortable or human.

In essence, the three studies we have presented are all modified Turing Tests in that they explore passing in one form or another (with Study 1 bearing the closest resemblance to Turing’s original concept). What our studies show is how intimately connected passing is to the social psychological framing of an interaction, and how the interface one communicates with affects the meaning of the situation from the point-of-view of interactants. In our own view, the results from Study 3 are at the same time the most profound and the least surprising. Seventeen of 20 people spoke face-to-face with an echoborg in a small room for 10-min and failed to develop even the slightest suspicion that they were interacting with the words of an artificial agent of some kind. They may have seen their interlocutor as strange, introverted, or even acting, but it did not cross their minds that who (or what) they were dealing with was part computer program. This makes sense in light of how we experience mundane human interaction, and implies that, given certain generic social psychological preconditions, an interlocutor’s capacity to produce sophisticated or even sensible syntax simply does not factor in to our categorizing them as a human being or as having a “mind.” That is to say, rather than taking these results as indicating the sophistication of chat bots, we take these results as indicating the importance of both the body and social psychological framing in social interaction.

### Future Research Applications

Creating human-like interfaces that totally override people’s awareness that they are interacting with something artificial remains a distant holy grail (Vogeley and Bente, 2010). In the interim, however, we can use echoborgs to approximate the conditions of a world in which machines are capable of passing the non-verbal and motor requirements of a Total Turing Test. This opens the doors to a new frontier of human–robot and human–agent interaction research.

Echoborgs can be used to further study uncanny valley phenomena. Most of the literature that has explored the uncanny valley has focused on motor behavior and physical resemblance as independent variables, as well as the effects different levels of participant engagement (passive vs. active) have on perceptions of agents (e.g., von der Pütten et al., 2011). Researchers have also, but to a lesser extent, looked at the role of phonetic quality in relation to the uncanny valley (e.g., Mitchell et al., 2011; Tinwell et al., 2011). Echoborgs enable us to study uncanny valley phenomena isolating dialogic capacity as an independent variable. Using echoborgs, we can see if an uncanny valley emerges when a spectrum of conversational agents ranging from the very poor (machine-like) to the very advanced (human-like) are communicated through a human speech shadower in unscripted face-to-face interactions.

Another possible avenue of research concerns the use echoborgs in comparative person perception studies. Experiments can be designed with conditions differentiated in terms of the interface through which participants communicate with a particular conversational agent (text interface, embodied conversational agent, echoborg, and so on). Researchers could then observe how the various interfaces shape aspects of the personality perceived by the participant, from minimal interfaces all the way up to a face-to-face human body.

A particularly enticing possibility for future research involves developing bots that simultaneously dictate words to a shadower while directing elements of the shadower’s motor behavior. In the echoborgs we have thus far constructed, the bot supplies the speech shadower with what to say while the shadower retains full control over their non-verbal functioning. We can imagine, however, developing a bot that delivered to the shadower’s left ear monitor words to speak while delivering basic behavioral commands (e.g., “smile,” “stand up,” “extend right hand for handshake”) to the shadower’s right ear monitor. This would grant the bot greater agency over the echoborg’s behavior.

The exciting opportunity opened up by echoborgs more generally is the opportunity to study human–computer interaction under the conditions of face-to-face human–human interaction. The problem for human–computer interaction research in general, and android science in particular, is that humans approach human–computer interaction differently from human–human interaction (as our own research shows). Human–human interaction triggers a huge range of complex phenomena, from identity dynamics to social emotions to basic taken-for-granted assumptions to an incredibly subtle intersubjective orientation to the other (Gillespie and Cornish, 2014). The echoborg method enables us to test conversational agents within face-to-face interaction scenarios, simultaneously pushing AI into a new domain and also to probing the full complexity of the human–human inter-*face*.

### Ethical Considerations

In exploring social contexts involving a covert echoborg, mild deception is required in order to preserve the participant’s belief that they are encountering an autonomous person. Careful experimental design (e.g., choice of conversational agents and shadowers, duration of interaction, communicative setting, etc.) and thorough piloting of procedures is strongly recommended so as to render participant distress unlikely. Participants should be exhaustively debriefed to gauge whether or not adjustments need to be made to the research procedures in order to avoid potential negative experiences. As a guideline, the debrief procedure in Study 3 involved asking the participant if they had any concerns regarding the ethics of the study as well as if they would object to a close friend or relative taking part in the same study under the same conditions. All participants said no to both questions. We can anecdotally report that all of our participants enjoyed taking part in our research, with many expressing positivity toward the echoborg concept during debriefing and linking their experiences with what they had seen in popular science fiction films.

### Limitations

Our studies were highly exploratory in nature. As such, various aspects of our investigations could have been more finely controlled. Though best attempts were made to standardize the body language of shadowers across all experimental trials, we did not make specific considerations for controlling certain behaviors (in particular, consistency of eye-contact). Moreover, the identity features of the shadowers (e.g., gender, ethnicity, age, and so on) may have produced unobserved effects on participants. We did not formally investigate such effects as they were not deemed to be of theoretical interest; however, we do acknowledge that questions regarding the relationship between the physical identity of the shadower and the social perception of the echoborg warrant future investigation. Sample sizes in our studies were relatively small due to practical constraints. Had our sample size for Study 3 been larger we might have been able to conduct a comprehensive comparison between the three chat bots used (Cleverbot, Rose, and Mitsuku). Also, we disclose that our choice of chat bots was based on prior familiarity with these programs.

We did not systematically analyze the effects audio latency may have had on participants’ experiences. The delay between interactant-utterances and echoborg-responses in the studies that involved participants speaking aloud to an echoborg certainly degraded the mundane realism of interactions to some degree. Minimizing this latency is a major research priority as we continue to refine the echoborg methodology. At the moment we face a trade-off between speed and accuracy: the use of a speed-typing third party (the minimal technological dependency model) slows the pace at which the conversational agent receives the words spoken by the interactant, yet better guarantees that the agent will process an accurate representation of the interactant’s words.

## Conclusion

This article has demonstrated the possibility and potential of echoborgs: human-bodied entities whose words (and potentially motor actions) are partially or completely determined by a computer program. Researchers can use echoborgs to study how people interact face-to-face with machine intelligence under the assumption that it is human. This methodology opens up a new paradigm for human–computer interaction research as to date people have interacted with computers, even sophisticated agents and highly lifelike androids, as machines (i.e., as things categorically different from real humans). Pairing a conversational agent with a human being to create an echoborg fundamentally transforms how people perceive and emotionally experience an in person encounter with social technology. Perhaps the most exciting takeaway from this initial examination of echoborgs is that under certain social psychological conditions echoborgs pass as fully autonomous human beings.

### Conflict of Interest Statement

The authors declare that the research was conducted in the absence of any commercial or financial relationships that could be construed as a potential conflict of interest.
